# Delayed transitions to adulthood and assisted reproduction: A study of educational differences in Spain

**DOI:** 10.1016/j.alcr.2025.100672

**Published:** 2025-04-26

**Authors:** Cristina Suero, Marie-Caroline Compans, Eva Beaujouan

**Affiliations:** a University of Vienna – Wittgenstein Centre for Demography and Global Human Capital ( IIASA, OeAW, University of Vienna), Austria; b Spanish National Research Council, Spain

**Keywords:** Assisted reproductive technologies, Spain, Transitions to adulthood events, ART outcomes, ART use, Educational differences

## Abstract

Transitions to adulthood are increasingly delayed in low-fertility countries, particularly among highly educated women, with significant implications for the timing of attempts to conceive and parenthood. Delayed child-bearing increases the risk of infertility and the reliance on assisted reproductive technologies (ART). Spain has experienced pronounced delays in transitions to adulthood alongside a substantial rise in ART use over recent decades. This research adopts a life course approach to examine the association between delayed transitions to adulthood, the likelihood of using ART, and the chances of achieving a live birth following ART, accounting for variations by age and educational attainment. Based on a sample of 12,930 women aged 24–55 from the 2018 Spanish Fertility Survey (SFS 2018), event-history analyses reveal that late first stable employment is associated with a lower likelihood of using ART, particularly for women without university education. Conversely, late housing independence and late coresidential partnership – up to the mid-30s – are linked to a higher likelihood of using ART. Among ART users, the likelihood of achieving a live birth decreases markedly with age, but declines less sharply for university-educated women. The timing of transitions to adulthood and the likelihood of achieving a live birth after ART are not related, except among women who left the parental home or entered a partnership particularly late, who are less likely to succeed. Overall, the findings suggest that ART offers limited capacity to mitigate the effects of delayed transitions to adulthood and fertility, especially for less educated women.

## 1 Introduction

Among the major changes in fertility observed in European countries since the 1970s, the steady delay in childbearing has received considerable attention. The age at first birth has increased by three to five years in most countries since 1970 (Nathan & Pardo, 2019), with a growing number of women becoming mothers in their forties, particularly in Southern European countries, such as Spain (Beaujouan, 2020). Transitions to adulthood – i.e., leaving the parental home, completing education, entering the labour market and forming a first co-residential union – are widely recognized as critical prerequisites to parenthood and are also occurring at later ages (Billari & Liefbroer, 2010; Gauthier, 2007). While the relationship is more complex than often assumed, the delay of these milestones tends to contribute to the postponement of pregnancy attempts and childbearing (Billari & Liefbroer, 2010; Buchmann & Kriesi, 2011; Kohler et al., 2002; Philipov et al., 2006). However, people are more likely to face age-related infecundity when trying to conceive later in life. Hence, along with a decline in births (Beaujouan, 2023), delayed childbearing appears to be driving both the growing demand for assisted reproductive technologies (ART) (Adamson et al., 2013) and the increasing number of ART births in Europe (Smeenk et al., 2023).

ART use can be seen as a way of compensating for the years lost due to delaying the transition to adulthood. However, postponing these transitions may not systematically and significantly lead to using ART to conceive. The use of ART often necessitates achieving a certain level of stability across various aspects of life. Furthermore, individuals may develop other goals that compete with childbearing and adjust their fertility aspirations downwards (Buhr & Huinink, 2017). They may forgo childbearing if they experience difficulties achieving pregnancy (Eijkemans et al., 2014), lack the necessary resources to afford ART and childbearing (Chambers et al., 2014), or simply choose not to use these technologies. An ART birth is also not necessarily systematic, as the effectiveness of ART declines with age (Hourvitz et al., 2009; Kocourkova et al., 2014; Leridon, 2017; Schmidt et al., 2012): women who delay transitions to adulthood tend to use ART later and therefore have a lower chance of achieving a live birth. While previous research has documented the importance of late transitions to adulthood for childbearing (Compans & Beaujouan, 2022; Mills et al., 2011), their relevance to ART uptake and outcomes has not been tested so far.

By adopting a life-course perspective, this paper examines whether the age at leaving the parental home, securing the first stable job, and forming a first co-residential partnership are connected to: 1) individual ART use; and 2) live birth outcomes after ART. We also explore how these dynamics vary by women´s educational attainment (Baizán et al., 2003; Billari et al., 2006, 2019; Gustafsson & Kalwij, 2006). Highly educated women tend to delay transitions to adulthood and their attempts to conceive than other educational groups, hence being more likely to use ART and at later ages. This may reduce their ART success rates. However, recent studies highlight that social and economic resources among this group may facilitate a birth through ART, despite their older ages (Alon & Pinilla, 2021; Goisis et al., 2020; Groes et al., 2017).

We focus on Spain, where transitions to adulthood occur particularly late (Bueno & Brinton, 2019; del Rey et al., 2023; Nishikido et al., 2022) and where the age at first birth has been consistently high for decades. In this country, assisted reproduction is widely available (Devolder & Borisova, 2022) and its use has increased considerably during the last years (Smeenk et al., 2023): almost 9 % of all births were attributed to ART in 2019, compared with 3 % on average in Europe (Smeenk et al., 2023). Spain, as a forerunner in these trends, can serve as an illustrative case for other countries where births and transitions to adulthood continue to be delayed. Using retrospective data from the recently released 2018 Spanish Fertility Survey, this study provides new insights into how ART may (or may not) mitigate the fertility consequences of late transitions to adulthood.

## 2 Background

### 2.1 Delayed transitions to adulthood and educational differences

Transitions to adulthood are commonly defined as the pivotal sociodemographic events that lead individuals to endorse social roles and achieve economic independence (Billari, 2004). They typically include completing education, leaving the parental home, entering the labor market, cohabiting with a partner for the first time and becoming a parent (Settersten, 2007). Traditionally, there were clear patterns of timing, occurrence, and sequence of these transitions (Buchmann & Kriesi, 2011; Marini, 1984; Rindfuss, 1991), with the timing of education completion typically influencing access to residential independence, professional career stability and partnership formation, and ultimately parenthood (Billari et al., 2006; Billari & Liefbroer, 2010; Kohler et al., 2002; Mills et al., 2011). Although these paths have become more diverse across European contexts over the past decades (Billari, 2004; Billari & Liefbroer, 2010; Buchmann & Kriesi, 2011; Mooyaart et al., 2022; Schoon, 2015), waiting to achieve professional and financial security, as well as a stable partnership, remains an important prerequisite for parenthood. Indeed, social perceptions of what a ‘good’ mother should be entail economic and emotional stability (Yopo Díaz, 2021a).

The timing of these events usually varies by social groups, with highly educated women delaying the transition to adulthood more than their less educated counterparts (Billari et al., 2019; Brons et al., 2017; Ferraretto & Vitali, 2023). Indeed, spending more time in formal education typically contributes to delaying subsequent events (Ní Bhrolcháin & Beaujouan, 2012). In addition to the delay associated with educational enrolment, highly educated women tend to face higher opportunity costs in raising children than less educated women (Becker, 1993; Gustafsson & Kalwij, 2006) and usually invest in their professional careers before starting a family (Mills et al., 2011). They also have different lifestyles and preferences than other social groups, which are thought to influence their fertility intentions and ideal timing at child-bearing (Badolato et al., 2024; Billari et al., 2019). However, less educated women also face more unstable working conditions, which may influence their age at trying to conceive and having children (Furstenberg, 2008).

### 2.2 Delayed transitions to adulthood and ART use

The ongoing tendency to postpone transitions to adulthood in high-income countries likely influences the delay in conception attempts. This delay may eventually extend to ages when fecundity is considerably reduced, prompting some individuals to seek medical help (Slauson-Blevins et al., 2013). Indeed, female patients in fertility clinics are often in their thirties, and around 56 % of women using IVF did so after age 34 in 2019 in Europe (Smeenk et al., 2023).

However, the connection between delayed transitions to adulthood and ART use involves complexities. Late transitions do not necessarily lead to the use of ART. For example, as most women are aware of the consequences of the biological clock (Yopo Díaz, 2021b), they may give up on having children or adjust their fertility desires downwards if they are single, have unstable partnership trajectories (Gray et al., 2013; Liefbroer, 2009), face challenges in trying to conceive (Brauner-Otto & Geist, 2018; Kuhnt et al., 2020), or if they pass the ideal age for having children. Women with delayed transitions to partnership or professional stability may also develop aspirations that compete with motherhood (Buhr & Huinink, 2017). Social norms about the “right” time to have children may also discourage them from pursuing parenthood after a certain age (Lazzari et al., 2024). Finally, women who have delayed these transitions may not have achieved a certain level of financial or professional stability and may be less able to afford the costs associated with starting a family, especially the less educated (Adserà, 2017).

As highly educated women tend to delay transitions to adulthood more often than less educated women and, therefore, attempt to conceive at later ages, they are expected to use ART more widely than other educational groups. This pattern has been shown in Norway (Goisis et al., 2020), Denmark (Groes et al., 2017), Finland (Raisanen et al., 2013), the United Kingdom (Dhalwani et al., 2013), and Spain (Alon & Pinilla, 2021). In addition to this postponement factor, other reasons may explain this higher propensity to use ART when experiencing infertility problems (Datta et al., 2016; Eisenberg et al., 2010). For instance, these women often have greater earning potential, which enables them to afford costly infertility treatments in contexts of constrained access to public treatment reimbursement (Chambers et al., 2014) and geographical proximity to fertility clinics (Lazzari et al., 2022). Highly educated women also tend to have greater knowledge about the techniques (Mahalingaiah et al., 2011) and hold more favorable perceptions of ART use (Szalma & Djundeva, 2019).

### 2.3 Delayed ART use and treatment outcomes

The probability of conceiving through assisted reproduction declines with age (Hourvitz et al., 2009; Leridon, 2017; Schmidt et al., 2012). As a result, women who use ART later because of delayed transitions to adulthood, typically highly educated women, may have lower chances of becoming mothers through ART (Goisis et al., 2023). However, recent research shows that highly educated women are more likely to achieve a pregnancy or a live birth than other groups in the United Kingdom (Goisis et al., 2020), Denmark (Groes et al., 2017), or the US (Lazzari & Tierney, 2023).

In contexts where treatments are partially or not subsidized, highly educated women often have greater resources to undergo multiple ART cycles (Chambers et al., 2014), access private clinics (Alon & Pinilla, 2021) or continue treatments (Mahalingaiah et al., 2011), potentially improving ART outcomes. Additionally, they generally enjoy better health over their lives compared to less educated women (Tain, 2003; van Dijk et al., 2024), and their greater flexibility in work schedules (Wallace, 2003) may facilitate the organization of clinic appointments, further potentially contributing to positive ART outcomes.

### 2.4 The Spanish case

Spain is an interesting case regarding delayed transitions to adulthood and ART use and outcomes. Like other Mediterranean European countries, it is characterized by particularly late transitions to adulthood (Lozano et al., 2024). This is often attributed to the country’s persisting economic uncertainty (Adsera, 2011; Bueno, 2020; Castro-Martín & Martín-García, 2013), especially among the young population, who often delay housing independence until meeting professional stability. The proportion of temporary employees among all employees aged 15–29 is 55 %, compared to the EU average of 36 % (Eurostat, 2019a). Consequently, the average age at leaving the parental home is among the highest in Europe, 29.8 in 2021 (Eurostat, 2021). This also influences co-residential partnerships, as a large proportion of individuals leave the parental home to live with a partner (del Rey et al., 2023). These delays, together with the scarcity of family public policies (Delgado et al., 2008; Lozano et al., 2024), contribute to the rise in very late fertility. Indeed, Spain holds the highest share of births after age 40 in the European Union – 10 % in 2019 (Eurostat, 2019b).

Parallel to these trends in delayed transitions to adulthood and reproduction, there has been a significant rise in the use of ART. According to the European Society of Human Reproduction and Embryology (ESHRE), the country boasts the highest number of ART clinics and the most significant increase in activity in Europe in 2018 (Wyns et al., 2022). Furthermore, 9 % of births in the country were attributed to ART in 2019 (Smeenk et al., 2023), a proportion that is expected to continue increasing in the coming years. Furthermore, among women aged 43 +, around 4.3 % of women living in Spain had used IVF over their lives (2018 SFS).

Assisted reproduction is regulated by Law 14/2006 on ART, which permits procedures without restrictions based on age, marital status, or sexual orientation. Treatments in public fertility clinics (hospitals) are almost free of charge. Public coverage is restricted to some specific requirements, such as an age limit (women under 40, with some exceptions),^1^ health status and parity (childless women),^2^ although these requirements may differ by region. The public system covers up to six intrauterine inseminations (IUI)^3^ and three IVF attempts. However, the public services have long waiting lists (Alon & Pinilla, 2021), prompting some to turn to the private system. In contrast, private clinics count on a large oocyte bank (Degli Esposti & Pavone, 2019), which is especially relevant for women using these treatments at late ages.

### 2.5 Hypotheses

This research aims to examine the relationship between delayed transitions to adulthood (such as housing independence, securing a first stable job, and entering a first co-residential partnership) and ART use, as well as the relationship between the age at which these events occur and ART outcomes.

First, women who delay transitions to adulthood may be more likely to use ART, as these transitions influence the age at which women attempt to conceive (Buchmann & Kriesi, 2011). Late conception attempts are associated with a higher probability of facing infertility issues, so *we expect age at experiencing transitions to housing independence, a first stable job and first co-residential partnership to be positively associated with the likelihood of ever using ART* (H1A). However, women who experience delayed transitions to adulthood may have reasons for not using assisted reproduction. For instance, they may not have achieved the financial, labor or partner stability required to raise a child or access ART. Additionally, they may give up on their desire to become mothers, adapting their fertility aspirations (Buhr & Huinink, 2017). Lastly, women who delay for too long may represent a selected group for whom using ART is less likely. Therefore, *late transitions to adulthood may be negatively or not associated with ART use* (H1B).

Highly educated women may be more likely to use ART. In addition to being more likely to postpone transitions to adulthood and pregnancy attempts, they usually have better financial resources and more flexible lifestyles (Goisis et al., 2023), which are compatible with ART use. Furthermore, highly educated women tend to seek medical help when needed, including reproductive treatments (Datta et al., 2016), and tend to have more open attitudes towards ART (Szalma & Djundeva, 2019). Additionally, delaying transitions to adulthood is uncommon among lower-educated women, so those who experience these delays may be a selected group, with fewer chances of meeting the requirements for using ART. Overall, *we expect delays to be more strongly associated with ART use among university educated than for those with lower levels of education* (H2).

Second, the extent to which Assisted Reproductive Technologies can compensate for fertility delays depends on their effectiveness, that is, whether women achieve a live birth as a result of treatment. Women who experience delayed transitions to adulthood may use ART at later reproductive ages, and the age at using ART has been negatively associated with lower treatment success rates (Hourvitz et al., 2009; Leridon, 2017; Schmidt et al., 2012). Consequently, *we expect the likelihood of achieving a live birth to be negatively associated with age at using ART* (H3). Additionally, recent research has indicated that highly educated women are more likely to achieve live births through ART. Therefore, *highly educated women who use ART may be more likely to achieve a live birth than their less educated counterparts, even if they use ART later* (H4).

## 3 Data and methods

### 3.1 Data and measures

We use the 2018 Spanish Fertility Survey (SFS),^4^ conducted by the National Statistics Institute (INE). The survey comprises a representative sample of 14,556 women living in Spain, aged between 18 and 55. It includes information on women’s reproductive, work and partnership histories until the time of the survey. Questions on reproductive history address whether women have ever tried to get pregnant, whether they have ever used infertility treatments, and their age at the first ART use. This research analyzes the likelihood of ever using in vitro fertilization (IVF) and intracytoplasmic sperm injection (ICSI) – which we refer to as assisted reproductive technologies (ART) (Zegers-Hochschild et al., 2017). The analytical sample only includes women aged 24 and over (n = 12,930), as by this age, women are likely to have completed their education.^5^ In this sample, 3.7 % of women have ever used ART. We explore the likelihood of using ART by controlling for information on women´s age at first stable job (permanent contract or self-employed with employees^6^), independent housing and co-residential partnership. We distinguished between four categories: the event has not happened (yet), the transition occurred between ages 24 and 29, between ages 30 and 34, and from age 35.^7^ We also control for the level of education, which distinguishes between non-university-educated women (ISCED 0–5) and university-educated women (ISCED 6–8). More than one-third of the sample have a university degree, and almost half of the women who use ART have that same educational attainment. Finally, we control for the women’s country of birth (Spain or other). The distributions of these indicators are displayed in Table 1.

Then, focusing on women who have ever used ART (n = 498), we assess the occurrence of at least one live birth after undergoing ART treatments, regardless of the number of cycles needed. Because we consider that women’s primary motivation in seeking medical help is to give birth to a child (Malizia et al., 2009) and the difficulties in separating ART from spontaneous conceptions, we do not only focus on ART births but also include natural conceptions during or after ART treatment.^8^ In addition to the variables indicating late transitions to adulthood, we control for age at ART use, parity when using ART, the time spent in treatment and the type of fertility clinic women went to (only public/private or both) (Table 1).

### 3.2 Methods

#### 3.2.1 Main analysis

We use event history analyses to assess: 1) the likelihood of ever using ART over the life course; and 2) the likelihood of achieving a live birth among those using ART. This allows us to account for right censoring among women interviewed before the end of their reproductive life.

In modelling the likelihood of ever using ART (1), a natural conception may be understood as a competing risk of using ART, as achieving a natural birth may reduce the likelihood of using ART afterwards. However, a natural birth does not necessarily prevent the event of interest – *ever* using ART – from occurring, especially for women who intend to have more than one child. Therefore, we use a special form of competing risk model that involves assigning time-dependent weights to individuals who experienced the competing event (Lambert, 2017). This keeps women who have a natural birth in the risk set, but with a lower analytical weight. This adjustment reflects the conditional probability of being censored due to the competing event – conceiving a child naturally (Lambert, 2017). The weight for individual *i* at time *t*_*j*_ is calculated as follows:

(1) $$w_{ij}=\{\begin{array}{c} \frac{{\hat{S}}_{c}(t_{j})}{{\hat{S}}_{c}(t_{e})}\;when\;experienced\;a\;natural\;birth\;at\;t_{e}<t_{j}\\ 0\;when\;censored\;before\;t_{j}\\ 1\;when\;still\;at\;risk\;\text{at}t_{j} \end{array}$$

with *S*_*c*_(*t*) the censoring distribution and t_e_ the time at experiencing the competing event – i.e., a natural birth. This is implemented using the *stcrprep* command in Stata.

To assess the likelihood of ever using ART, we use a flexible parametric version of the Fine and Gray model (Fine & Gray, 1999) that estimates cause-specific cumulative incidence functions using the stpm2 command in Stata. These models use cubic splines to estimate the hazard and survival functions^9^ (Lambert, 2017). The time of exposure begins at age 24 and ends at the time of the survey if the event of interest did not occur (right censoring), and at the age at initiating ART if women underwent such treatments. The baseline is the woman´s age.

Four models that estimate the use of ART are specified. M1 does not include the education variable, while M2 does; M3 includes an interaction between education and the event. An additional model M4 includes the three events and is presented in Appendix Table A1. All the models are weighted by *w*_*ij*_.

(2) $$\text{M1}:h_{k}(t|Event_{i},Z_{i})=\exp(\sum_{j=1}^{4}\gamma_{k,j}\times rcs_{j}(t)+\beta_{k}Event_{i,t}+\varphi Z_{i})$$

(3) $$\begin{array}{c} \text{M2}:h_{k}(t|Educ_{i},Event_{i},Z_{i})\\ \;=\text{exp}(\sum_{j=1}^{4}\gamma_{k,j}\times rcs_{j}(t)+\theta_{k}Educ_{i}+\beta_{k}Event_{i,t}+\varphi Z_{i}) \end{array}$$

(4) $$\begin{array}{l} \text{M3}:h_{k}(t|Educ_{i},Event_{i},Z_{i})\\ \;=\exp(\sum_{j=1}^{4}\gamma_{k,j}\times rcs_{j}(t)+\theta_{k}Educ_{i}+\beta_{k}Event_{i,t}+\theta_{k}Educ_{i}\\ \;\times \beta_{k}Event_{i,t}+\varphi Z_{i}) \end{array}$$

Where *h*_*k*_ is the estimated hazard of the event of interest - using ART(*k*), and *γ*_*k*_ are the coefficients of the restricted cubic splines(rcs), *β*_*k*_ is the coefficient of interest, *φ* is a vector of coefficients for the control vari-ables, and ***Z***_*i*_ is a vector of controls. *θ*_*k*_ are the coefficients for the level of education (*Educ*_*i*_). *Event*_*i*,*t*_ refers in alternance to the transition to a first stable job, leaving the parental home and first partnership. It is time-dependent and corresponds to an interaction between a binary variable that takes 0 until the interval at which individuals experienced the event and categorical variables indicating the occurrence and timing of each transition to adulthood. Thus, the variable is ‘Not occurred yet’ as long as the event has not happened and becomes equal to the age group in which the event happens once it occurs (24–29, 30–34 and 35 +). After excluding observations with missing values, these models are run among 12,930 women.

After analyzing the relationship between age at transitions and ART, we conduct a linear regression analysis to examine the connection between age at transitions and age at first ART use, presented in Table A2 in the Appendix. The results show a positive relationship between the age at using ART and both the age at the first stable job and the age at the first co-resident partner. Once this connection is established, we proceed to estimate the likelihood of achieving a live birth following ART treatments. With this aim, we use Cox regression models. The baseline refers to the time since beginning an ART treatment for the first time and ends at the time at having a live birth if the event occurred, and at the time of the survey or age 55 if the event did not occur (right-censoring). The model is described as follows:

(5) $$\begin{array}{l} \text{M5}:h(t)\\ \;=h_{0}(t)\times \text{exp}(\theta Educ_{i}+\beta Event_{i,t}+\varphi Age_{i,t}+\theta Educ_{i}\times \varphi Age_{i,t}+\gamma Z_{i}) \end{array}$$

where *h*_0_(*t*) is the baseline hazard function, *Age*_*i*,*t*_ is the age at using ART grouped into three categories (24–34, 35–39 and 40 +) and ***Z***_*i*_ is a vector of controls (country of birth, parity at treatment, duration of treatment and its quadratic term, and type of clinic women went to). Controls for transitions to adulthood events are included into the models in a second step. These models are run for 498 observations.

#### 3.2.2 Robustness checks and additional analyses

Several robustness checks and additional analyses are available upon request. Since some women have not experienced (yet) transitions to adulthood, we excluded those who did not experience any of the three life course events of interest in an additional analysis. Moreover, because some women may have started their relationship younger but delayed cohabitation until later ages, we conducted a similar analysis on a subsample of women for whom we have information on the date they started their relationship rather than the age at first co-residential partnership. Additionally, there may also be an overlap between different transitions to adulthood (del Rey et al., 2023). For this reason, we conducted an analysis including only women who did not simultaneously experience leaving the parental home and the first co-residential partnership. Finally, we compared models with different age-group categories for the baseline intervals and timing of transitions to adulthood (available upon request), as well as the timing of using ART. All these tests did not introduce remarkable changes compared to the main analyses presented.

## 4 Results

### 4.1 Timing and occurrence of transitions to adulthood by educational level

The descriptive statistics in Table 2 provide an insight into differences in the timing of transitions to adulthood and ART use by education in Spain, focusing on women aged 43–55 at the time of the survey. The mean age at leaving the parental home, first co-residential partnership and first birth is higher among university educated women (p < 0.001). There is no difference in the mean age of first stable employment (p > 0.05), although women with a non-university degree make this transi-tion less often (p < 0.001).

The use of ART is more prevalent among university educated women (7.6 % vs 2.9 %, p < 0.001). Among those who underwent treatments, the lower educated used them at an average age of 33.5 years, whereas university-educated women used them later (35.4 years, p < 0.001). Regarding the outcomes, among women who ever used ART, 53.5 % of university-educated women had a child, while 41.7 % of non-university-educated women did (p < 0.05). Women without university education spent longer time in treatments (27.7 months on average) than university-educated (22.3 months), although the difference is not statistically significant. Lastly, university-educated women are significantly more inclined to use private clinics (56.3 %) than the other group.

### 4.2 Delays in transitions to adulthood and use of ART

Models 1 in Table 3 present the relationship between each transition to adulthood and ART use, controlling for the country of birth. Women who have not (yet) secured a stable job are significantly less likely to use ART compared to those who achieved work stability at ages 24–29 (HR =0.61, p < 0.001, Model 1 A). The age at first job is negatively associated with the likelihood of using ART: women who achieve stability at ages 30–34 are somewhat less likely to use ART than those at 24–29, although the difference is not statistically significant (HR=0.82, p > 0.05). Those securing stability at age 35 or later show a much lower likelihood of using ART (HR=0.28, p < 0.01).

For housing independence, leaving the parental home at ages 30–34 increases the likelihood of ART use compared to leaving earlier (HR=1.39, p < 0.01, Model 1B). Conversely, women who have not yet co-resided with a partner are less likely to use ART (HR=0.73, p < 0.01, Model 1 C). Among those who have co-resided, women who experienced this transition at ages 30–34 are more likely to use ART than those who did so earlier (HR=1.39, p < 0.01). Similarly, women cohabiting for the first time after age 35 appear more likely to use ART, although the differences are not statistically significant.^10^ It is important to note that transitions after age 35 are rare in the sample (approximately 3 %), which may impact statistical power significance.

In sum, the timing of transitions to adulthood exhibits varying associations with the likelihood of using ART, depending on the specific event. Leaving the parental home and forming a first co-residential partnership at ages 30–34 appear to increase the likelihood of ART use (H1A). However, the effect becomes less clearly defined from age 35 onward, likely due to the limited number of cases in this age group. In contrast, the age at achieving a first stable job consistently shows a negative relationship with the likelihood of ever using ART (H1B).

Models 2 (Table 3) additionally control for educational attainment and slightly reduce the differences by age at each transition. All models show that women with a university degree are significantly more likely to use ART than women without a university education (HR=1.6, p < 0.01). The country of birth has no significant influence on ART use in all models.

Table 4 presents the hazard ratios for the interaction between age at transitions and education level. For the first stable job, the decline in ART use with increasing age at this transition is significantly steeper among women without a university education. For these women, experiencing a stable job after age 29 consistently reduces the likelihood of using ART, whereas the decline is less pronounced for women with a university education. In contrast, the positive association between the age at leaving the parental home or entering a first co-residential partnership and ART use is more pronounced among women without a university education compared to those with higher education, which contradicts H2.

### 4.3 Live births after using ART

Table 5 presents the results of Cox regression models estimating the hazard ratios for achieving a live birth following ART treatments. As expected, women initiating ART treatments between the ages of 35 and 39 are less likely to have a live birth (HR=0.65, p < 0.01) compared to those starting earlier, and the likelihood is even lower for those initiating treatment after age 39 (HR=0.44, p < 0.001). Additionally, women with a university degree have a higher likelihood of achieving a live birth than those without a university degree (HR=1.35, p < 0.05), even after controlling for age at treatment initiation, the type of clinic used, and the duration of treatment, supporting H3.

In the second model, we incorporate the ages at which transitions to adulthood events occur. This addition attenuates the effect of age at ART use on the likelihood of achieving a live birth, as the timing of these transitions is closely associated with the age at which ART is initiated. When accounting for the age at treatment initiation, only women who leave the parental home after the age of 35 and those who have not yet entered their first co-residential partnership show a significantly lower probability of having a child following ART.

**Fig. 1:**
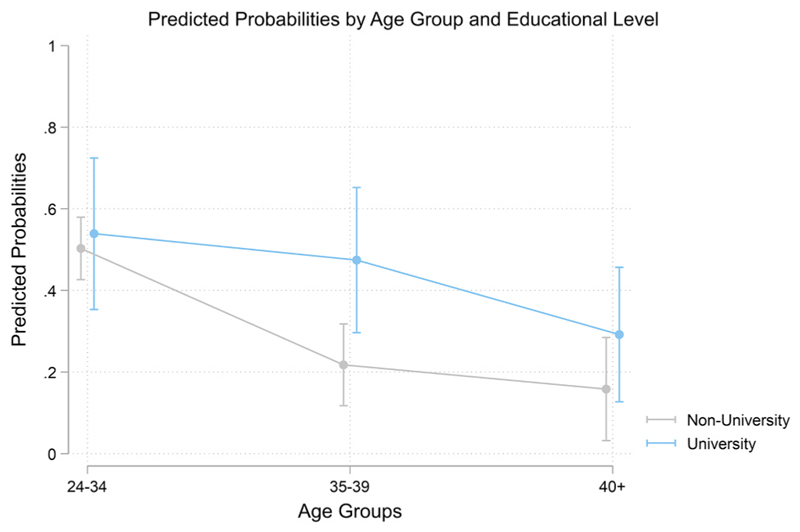
Results of the interaction between the level of education and the age at using ART for the first time on the probability of having a live birth after ART (Cox model). Author´s elaboration based on the 2018 SFS. Note: Results are reported as predicted hazards. CI: 95 %. The model controls for the time in treatment, birth parity and the country of birth.

Finally, Fig. 1 illustrates the interaction between age at starting ART treatments and education, presented as predicted probabilities. The results reveal that the decline in live births with increasing age at ART initiation is more pronounced for women without a university education. For women starting ART before the age of 35, the probability of having a live birth is relatively high: 0.54 for university-educated women and 0.50 for those without. However, for women initiating treatment between ages 35 and 39, the likelihood of a live birth declines markedly, particularly for women without a university education, dropping to 0.22. At these older ages, the education gap widens (0.26, p < 0.01). For women who begin ART after age 39, the probability of a live birth decreases further, with a steeper decline observed among university-educated women (0.29). However, at these ages, the differences in educational attainment become smaller and statistically insignificant (0.13, p > 0.05), as shown in Table A3 in the appendix.

## 5 Discussion

Transitions to adulthood—defined here as leaving the parental home, securing a stable job, and entering a first co-residential partnership—are increasingly delayed in Southern European countries, contributing to postponed childbearing (Billari & Liefbroer, 2010; Buchmann & Kriesi, 2011; Kohler et al., 2002; Philipov et al., 2006). Coupled with the age-related decline in fecundity (Te Velde et al., 2012), these delays may reduce the likelihood of achieving fertility aspirations. This study examines how the timing of transitions to adulthood relates to the use and outcomes of Assisted Reproductive Technology (ART). For ART to offset delayed childbearing, two conditions must hold: first, individuals who delay transitions to adulthood should be more likely to use ART; second, ART should lead to live births, even at older ages. Given that university-educated women often possess characteristics associated with higher ART uptake and success rates, this analysis also explores variations in ART use and outcomes by educational attainment.

The research focuses on Spain, a context characterized by exceptionally late transitions to adulthood (Bueno & Brinton, 2019; del Rey et al., 2023; Nishikido et al., 2022), and a significant increase in the use of ART over recent decades (Wyns et al., 2022), with these technologies being widely accessible. The country may serve as an illustrative case for understanding the implications of delayed transitions to adulthood and fertility in societies beginning to exhibit similar patterns, considering the potential of ART.

The findings show that the relationship between the age at experiencing transitions to adulthood and ART use varies by the type of transition. Women who leave the parental home and enter a first coresidential partnership in their early thirties are more likely to use ART, particularly non-university-educated women. Conversely, age at achieving a first stable job shows a consistently negative relationship with ART use, with the effect being stronger among women without a university degree. This disparity likely reflects the lower financial resources and reduced professional security faced by less-educated groups. Delays in achieving job stability, a widespread issue in in Spain (Bueno, 2020) appear to influence fertility timing (Lozano et al., 2024). These findings suggest that infertility treatments alone cannot offset for the reproductive impact of prolonged job precarity, especially for less-educated women. This dynamic may exacerbate socio-economic disparities in fertility opportunities.

The capacity of ART to offset the postponement of fertility associated with delayed transitions to adulthood is also influenced by its outcomes. This study suggests that delaying the transitions to adulthood may likely result in a later age of ART use, which in turn reduces the likelihood of achieving a live birth. There is limited evidence of a strong association between the timing of adulthood transitions and ART success: only women who left the parental home after age 35 or who have not yet entered a cohabiting relationship show a significantly lower likelihood of achieving a live birth after ART. Notably, educational differences play a role, with university-educated women experiencing a less pronounced age-related decline in live birth success. Although the differences diminish after age 40, university-educated women have a greater likelihood of achieving a live birth than their less-educated counterparts if they initiate ART treatments between the ages 35 and 39. As previous research has shown, greater job flexibility, better health status and more progressive attitudes toward ART use may contribute to higher ART success rates among university-educated women (Goisis et al., 2023; Tain, 2003; van Dijk et al., 2024; Wallace, 2003).

This study has some limitations. First, the data lacks retrospective information about fertility intentions making it difficult to determine whether women adjusted their fertility aspirations over their lives. Additionally, the dataset does not include information on attitudes toward ART or retrospective details about the partner’s job characteristics. Furthermore, there may be selection effects among women who postpone transitions to adulthood for extended periods, potentially influencing their likelihood of using ART—for example, health conditions or financial circumstances. However, the absence of retrospective data prevents us from adequately controlling for these factors.

Despite these limitations, this research provides valuable insights into the conditions under which ART can help women compensate for delayed fertility when experiencing late transitions to adulthood. The findings suggest that ART can partially offset the effects of delayed housing independence and partnership formation, particularly when these transitions occur by the early thirties. However, prolonged delays in achieving labor market stability may limit ART use, likely due to insufficient economic resources. The research also underscores significant social disparities in the capacity to use ART to recover lost time and achieve a live birth, even in a context where ART is publicly subsidized. The intersection of delayed life course transitions and the increasing reliance on reproductive technologies thus presents a mixed impact on society. On one hand, it offers opportunities for some women. On the other hand, it may amplify social inequalities in access to parenthood. This trend raises questions about reproductive rights and calls for policies addressing these emerging societal challenges.

## Supplementary Material

Appendix

## Figures and Tables

**Table 1:** Descriptive statistics of the variables.

	All women	Women using ART
First stable job (%)		
Not occurred yet	41.9	22.7
Under 30	43.8	61.4
30–34	6.6	8.9
35 +	7.8	7.1
Leaving the parental home (%)		
Not occurred yet	19.8	11.2
Under 30	69.2	67.0
30–34	8.6	17.5
35 +	2.4	3.9
First coresidential partner (%)		
Not occurred yet	40.0	24.3
Under 30	48.1	50.2
30–34	8.7	18.4
35 +	3.2	7.2
Age at ART use (%)		
Under 30		19.4
30–34		36.2
35 +		44.3
Used ART (%)	3.7	
Live birth following ART (%)		58.8
Type of clinic used (%)		
Public and private or only private		73.7
Only public		26.3
Born in Spain (%)		
No	18.7	9.6
Yes	81.3	90.4
Parity (at time of the survey/ at using IVF)		
(%)		
0	38.4	86.4
1	22.9	10.9
2 +	38.7	2.8
Educational level (%)		
Non-university	64.9	52.2
University	35.1	47.8
N	12,930	498

Note: Authors´elaboration based on the SFS 2018. Sample: Women aged 24 or over

**Table 2:** Mean age at transitions to adulthood (in years) and descriptive statistics (distribution in %) by level of education, statistical test of the difference.

	Non- University	Chi-2 / *t-*test	University
**All women aged 43 or more**			
Never had a stable job (%)	44.3	* **	19.6
Mean age at first stable job	28.1		28.2
Never left the parental home (%)	15.5	* **	11.2
Mean age at leaving the parental home	24.5	* **	27.3
Never lived with a partner (%)	32.5	* **	30.8
Mean age at first co-resident partner	25.6	* **	29.3
Mean age at first child	27.5	* **	32.4
Having ever used IVF/ICSI (%)	2.9	* **	7.6
N (unweighted)	4509		1829
**Women using ART (from 24 on)**			
Live birth after IVF/ICSI (%)	41.7	*	53.5
Mean age at first IVF use	33.5	* **	35.4
Mean first treatment duration (months)	27.7		22.3
Use of private clinics (%)	26.9	* **	56.3
N (unweighted)	249		249

Notes: Sample: Women aged 43 +. Results are weighted. Mean ages at events only include women who experienced them. The test column shows the statistical significance of the difference between the non-university educated and the university educated (2-sided test), using chi-2 tests for categorical variables and *t*-test for averages.

* p < 0.05

* * p < 0.01

* ** p < 0.001.

**Table 3:** Results of the flexible parametric models with weights accounting for competing risks estimating the use of ART.

	First stable job			Leaving parental home			First co-resident partner	
	Model 1 A	Model 2 A		Model 1B	Model 2B		Model 1 C	Model 2 C
***Timing of the event (t)***								
**Not happened yet**	0.609 (0.06) * **	0.667 (0.07) * **		0.882 (0.12)	0.922 (0.13)		0.734 (0.08) * *	0.724 (0.08) * *
**24–29 (ref.)**								
**30–34**	0.815 (0.15)	0.792 (0.15)		1.392 (0.18) * *	1.307 (0.17) *		1.393 (0.18) * *	1.272 (0.17) +
**35 +**	0.276 (0.11) * *	0.289 (0.11) * *		0.793 (0.23)	0.765 (0.23)		1.311 (0.28)	1.208 (0.26)
***Educational level***								
**Non-University (ref.)**								
**University**		1.596 (0.15) * **			1.677 (0.16) * **			1.660 (0.16) * **
***Country of birth***								
**Spain (ref.)**								
**Other**	0.989 (0.16) * **	1.019 (0.17)		0.936 (0.15)	0.977 (0.16)		0.946 (0.16)	0.988 (0.16)
***Spline function coefficients***								
**rcs1**	4.911 (1.75)	4.912 (1.75) * **		4.921 (1.75) * **	4.921 (1.75) * **		4.913 (1.75) * **	4.909 (1.75) * **
**rcs2**	0.779 (0.42)	0.780 (0.42)		0.771 (0.41)	0.771 (0.41)		0.766 (0.41)	0.767 (0.41)
**rcs3**	0.129 (0.16) +	0.127 (0.16) +		0.133 (0.16)	0.131 (0.16)		0.138 (0.17)	0.136 (0.17)
**rcs4**	32.472 (28.38) * **	33.015 (29.02) * **		31.840 (27.61) * **	32.377 (28.25) * **		30.554 (26.35) * **	31.066 (26.94) * **
***Constant***	0.000 (0.00) * **	0.000 (0.00) * **		0.000 (0.00) * **	0.000 (0.00) * **		0.000 (0.00) * **	0.000 (0.00) * **
**N**	12,930							

Note: Authors´elaboration based on the 2018 SFS. The models attribute time-dependent weights to individuals who experienced the competing event – natural birth. Results are reported as hazard ratios. Standard deviations are between brackets.

* p < 0.05

* * p < 0.01

* ** p < 0.001

**Table 4:** Results of the interactions between age at experiencing events and educational level, by transition (flexible parametric models with weights accounting for competing risks).

	Stable job			Leave the parental home			First partner	
	Non-university	University		Non-university	University		Non-university	University
**Not happened yet** **24–29 (ref.)**	0.638 (0.09) * *	0.693 (0.11) *		0.812 (0.16)	1.078 (0.22)		0.719 (0.12)	0.727 (0.12)
**30–34**	0.506 (0.18) +	0.983 (0.22)		1.416 (0.28) +	1.244 (0.21)		1.414 (0.28)+	1.186 (0.20)
**35** +	0.220 (0.13) * *	0.371 (0.19) +		0.731 (0.33)	0.793 (0.31)		1.146 (0.40)	1.239 (0.33)

Notes: Author´s elaboration based on the 2018 SFS. Results are reported as hazard ratios. Standard deviations are between brackets.

+ p < 0.1

* p < 0.05

* * p < 0.01

* ** p < 0.001

**Table 5:** Results of the Cox model estimating the probability of having a live birth after ART.

	HR	HR
*Age at ART*		
24–34 (ref.)		
35–39	0.653 (0.09) * *	0.707 (0.10) *
40 +	0.437 (0.09) * **	0.530 (0.15) *
*Educational level*		
Non University (ref.)		
University	1.353 (0.17) *	1.289 (0.16) *
*Age at first stable job*		
Not happened (yet)		0.921 (0.12)
24–29 (ref.)		
30–34		0.846 (0.22)
35 +		0.455 (0.27)
*Age at leaving parental home*		
Not happened (yet)		0.970 (0.184)
24–29 (ref.)		
30–34		0.902 (0.22)
35 +		0.196 (0.15) *
*Age at first co-residential partner*		
Not happened (yet)		0.603 (0.10) * *
24–29 (ref.)		
30–34		1.043 (0.24)
35 +		1.336 (0.46)
*Type of clinic used*		
Public and private or only private (ref.)		
Only public	1.083 (0.15)	1.055 (0.15)
*Time in treatment*	0.959 (0.01) * **	0.958 (0.01) * **
*Country of birth*		
Native (ref.)		
Foreigner	0.789 (0.18)	0.738 (0.17)
*Parity*		
Parity 0 (ref.)		
Parity 1 +	0.772 (0.18)	0.817 (0.19)
N	498	498

Notes: Author´s elaboration based on the 2018 SFS. Results are reported as hazard ratios (HR). Standard deviation are between brackets.

* p < 0.05

* * p < 0.01

* ** p < 0.001

## Footnotes

1 Women under 38 if using the partner´s gametes.

2 For couples, ART is covered if they do not have any child in common.

3 For women aged up to 37. Four attempts if the sperm comes from the partner.

4 More information about the survey can be found online: https://www.ine.es/uc/xtorCkFs

5 Only four women in the survey have used ART before this age.

6 We include self-employed individuals with employees to differentiate them from those without employees, as the former generally indicates greater stability in most cases.

7 An additional analysis using different age categories can be found in Table A3 in the Appendix.

8 Only five women reported a natural birth after using ART treatments.

9 We cannot use the simpler Proportional Hazards models because the proportional hazards assumption would be violated.

10 Results remain similar when we test the effect of the timing of the start of a first relationship instead of the cohabitation, although on a smaller sample size.
