# Supplementary material for: Delayed transitions to adulthood and assisted reproduction: A study of educational differences in Spain
Source: Adv Life Course Res. Author manuscript; Available in PMC 2025 May 12. (PMC7617658; doi:10.1016/j.alcr.2025.100672)
Supplement: Appendix [file EMS205274-supplement-Appendix.pdf]

Appendix

Table A1
Results of the flexible parametric models with weights accounting for competing risks estimating the use of ART by age at the first stable job, leaving the parental home and first co-residential partnership

	Model 1D	Model 2D
First stable job (t)		
**Not happened yet**	0.614 (0.06) * **	0.665 (0.07) * **
**24–29 (ref.)**		
**30–34**	0.787 (0.15)	0.775 (0.15)
**35 +**	0.283 (0.11) * *	0.296 (0.11) * *
Leaving parental home (t)		
**Not happened yet**	0.974 (0.14)	1.018 (0.15)
**24–29 (ref.)**		
**30–34**	1.244 (0.21)	1.244 (0.21)
**35 +**	0.738 (0.24)	0.753 (0.24)
First co-resident partner		
**Not happened yet**	0.737 (0.09) *	0.728 (0.09) * *
**24–29 (ref.)**		
**30–34**	1.210 (0.20)	1.131 (0.19)
**35 +**	1.433 (0.34)	1.326 (0.31)
Educational level		
**Non University (ref.)**		
**University**		1.543 (0.15) * **
Country of birth		
**Spain (ref.)**		
**Other**	1.020 (0.17)	1.050 (0.17)
Spline function coefficents		

(*continued on next page*)

Table A1 (*continued*)

	Model 1D	Model 2D
**rcs1**	4.902 (1.74) * **	4.902 (1.74) * **
**rcs2**	0.782 (0.41)	0.782 (0.42)
**rcs3**	0.133 (0.16)	0.131 (0.16)
**rcs4**	30.844 (26.80) * **	31.439 (27.47) * **
Constant	0.000 (0.00) * **	0.000 (0.00) * **
**N**	12,930	

Notes: Author's elaboration based on the 2018 SFS. Results are reported as hazard ratios (HR). Standard deviation are between brackets. Note:
+ p < 0.1,
* p < 0.05,
* * p < 0.01,
* ** p < 0.001.

Table A2
Results of the linear regression predicting the age at using ART by ages at experiencing transitions to adulthood among women who have ever used ART

	Linear regression
First stable job (t)	
**Not happened yet**	−0.067 (0.05)
**24–29 (ref.)**	
**30–34**	0.088 (0.07)
**35 +**	0.208 (0.08) *
Leaving the parental home (t)	
**Not happened yet**	−0.012 (0.07)
**24–29 (ref.)**	
**30–34**	0.098 (0.07)
**35 +**	0.071 (0.11)
First co-resident partner	
**Not happened yet**	0.289 (0.05) * **
**24–29 (ref.)**	
**30–34**	0.286 (0.07) * **
**35 +**	0.619 (0.09) * **
Constant	0.260 (0.03) * **
**N**	498

Notes: Author's elaboration based on the 2018 SFS. Standard deviation are between brackets.
+ p < 0.1,
* p < 0.05,
* * p < 0.01,
* ** p < 0.001.

Table A3
Results of the flexible parametric models with weights accounting for competing risks estimating the use of ART, and with different age categories

	First stable job		Leaving parental home		First co-resident partner	
	**Model 1 A***	**Model 2 A***	**Model 1B***	**Model 2B***	**Model 1 C***	**Model 2 C***
Timing of the event (t)						
**Not experienced (yet)**	0.620 (0.07) * **	0.668 (0.07) * **	0.898 (0.13)	0.915 (0.13)	0.760 (0.10)	0.719 (0.09) * *
**24–27 (ref.)**						
**27–29**	1.095 (0.17)	1.005 (0.15)	1.062 (0.14)	0.976 (0.13)	1.101 (0.15)	0.983 (0.13)
**30–32**	0.791 (0.18)	0.760 (0.17)	1.665 (0.23) * **	1.521 (0.21) * *	1.727 (0.25) * **	1.515 (0.22) * *
**33–36**	0.854 (0.24)	0.825 (0.24)	0.677 (0.21)	0.625 (0.19)	0.641 (0.19)	0.556 (0.17) +
**37 +**	0.195 (0.10) * *	0.202 (0.10) * *	0.747 (0.27)	0.708 (0.26)	1.552 (0.36) +	1.377 (0.32)
Educational level						
**Non-University (ref.)**						
**University**		1.020 (0.17)		0.981 (0.16)		0.995 (0.16)
Country of birth						
**Spain (ref.)**						
**Other**	0.991 (0.16)	1.595 (0.15) * **	0.950 (0.16)	1.677 (0.16) * **	0.956 (0.16)	1.669 (0.16) * **
Spline function coefficients						
**rcs1**	4.906 (1.75) * **	4.909 (1.75) * **	4.921 (1.75) * **	4.921 (1.75) * **	4.914 (1.75) * **	4.911 (1.75) * **
**rcs2**	0.774 (0.41)	0.775 (0.41)	0.777 (0.41)	0.776 (0.41)	0.774 (0.41)	0.774 (0.41)
**rcs3**	0.132 (0.16) *	0.130 (0.16)	0.130 (0.16) +	0.128 (0.16) +	0.134 (0.16)	0.132 (0.16)
**rcs4**	31.860 (27.86) * **	32.426 (28.52) * **	32.339 (28.09) * **	32.872 (28.73) * **	31.295 (27.01) * **	31.836 (27.64) * **
Constant	0.000 (0.00) * **	0.000 (0.00) * **	0.000 (0.00) * **	0.000 (0.00) * **	0.000 (0.00) * **	0.000 (0.00) * **
**N**	12,930					

Note: Authors' elaboration based on the 2018 SFS. The models attribute time-dependent weights to individuals who experienced the competing event – natural birth. Results are reported as hazard ratios. Standard deviations are between brackets.

+ p < 0.1
* p < 0.05
* * p < 0.01
* ** p < 0.001

Table A4
Results of the interaction between educational attainment and age at using ART for the first time on the probability of having a live birth after ART (Cox model)

	Predicted probabilities
(1) 24–34 Non Uni	0.503 (0.04) * **
(2) 23–34 Uni	0.539 (0.09) * **
(3) 35–39 Non Uni	0.218 (0.05) * **
(4) 35–39 Uni	0.474 (0.09) * **
(5) 40 + Non Uni	0.158 (0.06) *
(6) 40 + Uni	0.292 (0.08) * *
	Simple differences
2–1	0.036
4–3	0.257 *
6–5	0.134
6–4	−0.182 +
4–2	−0.065
5–3	−0.059
3–1	−0.285 * **
5–1	−0.344 * **
6–2	−0.247 * *
	Double differences
(6−2) - (5−1)	0.097
(4−2) - (3−1)	0.221 *

Notes: Authoŕs elaboration based on the 2018 SFS. Results are reported as predicted probabilities. Standard deviations are between brackets. + p < 0.1 * p < 0.05 * * p < 0.01 * ** p < 0.001. The model controls for time in treatment, birth, parity and country of birth.
